# Diphtheritic Inflammation of the Pharynx and Tonsils

**Published:** 1849-06

**Authors:** Henry A. Ramsay

**Affiliations:** Raysville, Geo.


*Diphtheritic Inflammation of the Pharynx and Tonsils.* By HENRY A. RAMSAY, M. D., Raysville, Geo.

So much has recently been written upon diphtheritic affections of the throat, that I should forbear to offer this article to the profession, had not the disease here assumed some features of deviation from its usual course at other points. It is not my design to enter into a prolix argument upon the pathological rationale of the epidemic in question, but simply to present the readers of this paper with a truthful synopsis of what I saw at the bedside of the patient. Without descending farther into unessential details, I will remark that the popular cognomina of the disease among us are *sore throat, swelled tonsils, influenza, or quinsy.* An attack is usually preceded by a rigor or chill of more or less intensity; but this is not the invariable mode of premonition. We have often seen it preceded, and it is not unusual, by a pain in the *leg, arm, toe, finger, ear* or *abdomen.* With this premonitory outline, the disease assumes two distinct stages, viz. an acute; and typhoid, or malignant. After the supervention of the above symptoms, the acute stage is ushered in by sore throat, dryness of the throat, some pain in the head, some suffusion of tears, with a profuse discharge from the nose; the pulse is full and rather inclined to hardness; the skin is harsh and dry; the eyes are red, the tonsils, pharynx, fauces, etc., are swollen, and covered as far as the eye can observe, with patches of a membranous character, resembling ulcerations; deglutition is attended with some degree of difficulty; pultaceous substances being swallowed with more facility than fluids; there is usually a slight cough and discharge of a tough, viscid phlegm; the tongue is covered with a dirty white coat, almost instinctively impressing one with the idea of malignancy, or obstinacy; the parotid and submaxillary glands are faintly swollen; the limbs ache, and the patient complains of weariness, of indisposition, and of a burning about the throat, with thirst. When his vocal organs are much exercised, he craves water, but cannot swallow it with ease; his respiration is frank and open, but you can discern a nasal twang when he speaks; his appetite is perverted, and he seldom eats.

These symptoms continue for an indefinite period, not longer usually than four or five days, when the malignant stage advances, by almost irresistible and insensible gradations; the tongue assumes a dark colour; almost a *charcoal* appearance. This appearance of the tongue exists sometimes in the first stage of the disease. The patient mopes about almost lifeless; he is stupid, dull, and despondent; his cutaneous discharge is cool, sticky, and unpleasant to the smell; his pulse is feeble and languid; his bowels, in the first stage constipated, now become loose, and his discharges fetid; he has loss of appetite, deafness, pain in the ears; his limbs ache and grow stiff; his mouth gets sore and bleeds, and, to use a familiar term, it feels "nasty" to him; his throat is not offensive, nor is his deglutition more impaired than in the preceding stage. He will remain possibly in this state for ten or twelve days, when he would surely die, if no plan of treatment were adopted. The above is a faithful description of the course of the disease here, so far as my professional limits extend, and I am led to conclude it is the same elsewhere, from what I have gathered from other creditable sources. The malady here is certainly very malignant, not exceedingly fatal, however, when we reflect for a moment upon its severity. The disease is not confined to any particular period of life. Young and old are alike the victims of its attack; our black population, however, are more frequently its subjects. The prognosis I regard as generally favourable. Children die usually in the first stage; adults in the second.

Most of the adults who have died were attacked with a pain in some one of the limbs; cerebral inflammation soon supervenes, and the patient sinks rapidly under its effects.

The causes of this affection are somewhat obscure. Many believe it of a specific origin, others think it generated by atmospheric fluctuations. It is common for "Doctors" to differ, and a diversity of sentiment is a prolific source for a diversity of thought; consequently a contrariety of opinion has a good effect upon inductive minds. This conflict of sentiment has induced me to ascribe this disease, at least so far as this country is concerned, to a malarious origin; I am aware this is a mooted point in reference to many diseases, but my observation leads me to adopt this conclusion in reference to this disease, though I am not disposed in this paper to enter the arena of argument upon these unsettled points.

It will not be amiss to advance one proposition which, if it be tenable, and I cannot for a moment doubt it, is of increasing interest to the farmers of this county; it is that the malarious origin of this and many other diseases in this country may be based upon the following facts:

We are undergoing an agricultural regeneration in Georgia; our farmers are making large quantities of manure to retrieve their worn out hills; they are clearing up their small branch bottoms, hitherto uncared for and unthought of; they are felling from sheer necessity large forests, to open fresh fields of agriculture, and in a large majority of instances the timber is not cleanly cut, but left to *decompose*, to *rot*. All these circumstances conspire to make the sources of malaria prolific, and consequently to generate disease. The manure lots and beds are made, in nine cases out of ten, very injudiciously, near the dwelling houses, and it is seldom they are exempt from a nauseous stench. Now I have found it an unfailing rule, that those farmers who had the filthiest lots nearest their houses, had most cases of sore throat, particularly if the hands were engaged in scraping the lots or hauling the manure.

It is customary here in the fall, to draw into the lots a large quantity of leaves, cotton stalks and straw; this is permitted to rot during the winter and summer; hence the large number of cases of chill and fever we have had for the last few years.

So long as these pernicious practices are persisted in, so long will we have epidemics, intermittent and remittent fevers, in this country. But you ask me the remedy? It is so simple, that I will reply by propounding a simple question. If a small branch was inundating a fine plat of ground which you wished to preserve, how would you remedy it? You will readily answer, ditch it, turn it, *move it*. Move your lot, and that, too, at such a distance that its influence cannot affect you.

With merely remarking that I am induced to favour the idea that this disease is contagious, I will pass on to the treatment.

In the treatment of this affection, simplicity is our guiding star; and when I say simplicity, I mean an avoidance of every thing *harsh* or *drastic*. In the first stage, we use emetics of a mild kind; they relieve the throat of that sense of suffocation which sometimes exists, from the accumulation of tough phlegm. They also appear to us to exert a good influence in assisting the modification of the secretions of the mucous membrane of the throat, and not unfrequently cast off those membranous patches observed upon the tonsils and fauces. For all practical purposes, we know of no emetic equal to the common table salt, exhibited in a warm solution ; we prefer it to all others for its mildness and expeditious of action. Next to this, we prefer a combination of mustard and ipecac, or the latter alone. We prescribe these emetics through the whole course of the disease ; and so perfectly convinced are we of its propriety, that we challenge a comparison of success in an equal number of cases with any man.

After emesis, we prescribe a dose of castor oil, or sulph. magnesia, and pepper tea, every day or every other day, as occasion may require. As a local application to the throat internally, we use a warm solution of alum or vinegar and warm water ; either has a happy effect. The nitrate of silver and kreosote, so often prescribed in diphtheritic diseases, did not succeed well in our hands. As an external remedy to the throat, we conceive the ordinary pepper poultice, or onion poultice, far superior to any other ; indeed, we consider it of unequivocal advantage in every case. Scarification of the tonsils is our magnum remedium in all cases of throat disease, where the tonsils are much enlarged or swollen. As a therapeutic application, we doubt the efficacy of blisters. We have seen them the cause of immense suffering and pain. The suggestion of blood-letting we should deem detrimental, and not at all to be entertained.

The treatment of the second stage must be essentially modified in many respects. When the tongue is black, the circulation languid and dull, we know of no remedy equal to blue pill and cayenne pepper, to restore the vital energies. We do not mention the quantity, as the judgment of the practitioner can best determine the quantity necessary in any case. In combination with this, we use a warm pediluvium every night, and sometimes during the day. We also direct the occasional use of porter or wine, if there be much prostration.

Blisters in this form of the affection have a deleterious effect, and should be strongly reprobated. While we regard blue pill as an admirable agent in the malignant form, we do not wish to be considered as endorsing its use, or that of any other mercurial, in the first stage.

The preparations of cinchona have never had any good effect in our hands; they generally produce a feverish state of the system, ending in depression.

